# Reduced Placental Transfer of Antibodies Against a Wide Range of Microbial and Vaccine Antigens in HIV-Infected Women in Mozambique

**DOI:** 10.3389/fimmu.2021.614246

**Published:** 2021-03-03

**Authors:** Selena Alonso, Marta Vidal, Gemma Ruiz-Olalla, Raquel González, M. Nelia Manaca, Chenjerai Jairoce, Miquel Vázquez-Santiago, Reyes Balcells, Anifa Vala, María Rupérez, Pau Cisteró, Laura Fuente-Soro, Marta Cova, Evelina Angov, Arsenio Nhacolo, Esperança Sevene, John J. Aponte, Eusebio Macete, Ruth Aguilar, Alfredo Mayor, Clara Menéndez, Carlota Dobaño, Gemma Moncunill

**Affiliations:** ^1^ISGlobal, Hospital Clínic - Universitat de Barcelona, Barcelona, Spain; ^2^Centro de Investigação em Saúde de Manhiça (CISM), Maputo, Mozambique; ^3^U.S. Military Malaria Vaccine Program, Walter Reed Army Institute of Research (WRAIR), Silver Spring, MD, United States; ^4^Department of Physiologic Science, Clinical Pharmacology, Faculty of Medicine, Eduardo Mondlane University, Maputo, Mozambique

**Keywords:** antibody, maternal antibodies, placental transfer, HIV, pathogens, malaria, IgG, IgG subclasses

## Abstract

Transplacental transfer of antibodies is essential for conferring protection in newborns against infectious diseases. We assessed the impact of different factors, including gestational age and maternal infections such as HIV and malaria, on the efficiency of cord blood levels and placental transfer of IgG subclasses. We measured total IgG and IgG subclasses by quantitative suspension array technology against 14 pathogens and vaccine antigens, including targets of maternal immunization, in 341 delivering HIV-uninfected and HIV-infected mother-infant pairs from southern Mozambique. We analyzed the association of maternal HIV infection, *Plasmodium falciparum* exposure, maternal variables and pregnancy outcomes on cord antibody levels and transplacental transfer. Our results show that maternal antibody levels were the main determinant of cord antibody levels. Univariable and multivariable analysis showed that HIV reduced the placental transfer and cord levels of IgG and IgG1 principally, but also IgG2 to half of the antigens tested. *P. falciparum* exposure and prematurity were negatively associated with cord antibody levels and placental transfer, but this was antigen-subclass dependent. Our findings suggest that lower maternally transferred antibodies may underlie increased susceptibility to infections of HIV-exposed infants. This could affect efficacy of maternal vaccination, especially in sub-Saharan Africa, where there is a high prevalence of HIV, malaria and unfavorable environmental factors.

## Introduction

Each year, 2.6 million deaths occur during the neonatal period, with infectious diseases being the leading cause of mortality, particularly in low-income countries (1, 2). Newborns are highly vulnerable to pathogens due to their functional immunological differences from adults as a result of living in a semi-allogeneic sterile environment, where exposure to microbial antigens is limited (3–6). For example, microorganisms such as respiratory syncytial virus (RSV) cause no or mild disease in adults but induce acute bronchiolitis, viral pneumoniae, and croup in infants, with those between 2 and 6 months of age at the highest risk, especially in low-income countries (7, 8).

Vaccination is among the most cost-effective public health measures worldwide (9), and regions with high rates of infant morbidity and mortality like sub-Saharan Africa benefit from the implementation of the Expanded Program of Immunization (EPI) (10). Nevertheless, acquisition of immunity from vaccination is not immediate and vaccines are not available for all infectious diseases. At present, only three vaccines are being administered at birth in some countries: Bacillus Calmette-Guérin (BCG), hepatitis B virus (HBV), and oral polio vaccine (OPV) (11–13). Therefore, newborns mostly rely on the protection elicited by maternal antibodies transferred across the placenta, which provide passive immunity against common pathogens (14).

Transplacental transfer of antibodies occurs *in utero* and it is facilitated by neonatal fragment crystallizable (Fc) region receptor (FcRn), expressed in the human syncytiotrophoblast (15, 16). Only IgG is transferred across the placenta with the highest rate occurring during the third trimester of pregnancy (17), although some studies suggest that maternal IgE may also be transferred to the fetus as IgG/IgE complexes (18). IgG subclasses have different affinities for the FcRn receptor leading to differences in the efficiency of transfer (19); classically it was stated that the greatest transport occurs for IgG1, followed by IgG4, IgG3, and finally IgG2 (20), although a recent update on transplacental transfer of IgG subclasses showed that it is different depending on the antigen and study populations (21).

To be effective, the transferred IgG must reach protective levels after birth. Maternal immunization is a valuable strategy to prevent newborn infections, ensuring a sufficient transfer of protective antibodies to the neonate (22, 23). Maternal vaccination against tetanus, pertussis, and influenza has been implemented in many populations and has been effective at protecting young infants from these pathogens (24–26), and could be used to protect newborns from RSV (27). However, it has been reported that IgG placental transfer and cord levels could be affected by some factors such as maternal antibody concentrations, gestational age, placental integrity, maternal infections, Fc binding strength, and the antigen specificity (16, 28–32), although the effects are not consistent among studies. Placental malaria (PM) has been shown to reduce transplacental transfer of antibodies against tetanus, measles, *Streptococcus pneumoniae* (*S. pneumoniae*), herpes simplex virus type 1 (HSV-1), RSV and varicella-zoster virus (VZV) (28, 33–35). However, other studies have shown no impact of PM on transplacental transfer of tetanus, *S. pneumoniae, Haemophilus influenzae type b* (*Hib*), diphtheria, measles, or RSV antibodies (16, 33, 35–38). The effect of maternal HIV infection is also controversial. Some studies demonstrated that HIV infection leads to a reduction of the transplacental transfer of *Hib*, diphtheria, pertussis, pneumococcus, measles, tetanus, and *Plasmodium falciparum* (*P. falciparum*) specific antibodies (28, 29, 39–45), but others have shown no effect (29, 33, 38, 41, 42, 46). Those studies had several limitations, as the numbers of antigens tested and HIV-infected women included in the analyses were low. Moreover, the information regarding IgG subclasses is scarce (41, 45, 46). Therefore, the effect of HIV and malaria on transplacental transfer of antibodies, particularly IgG subclasses, is still not clear.

In our study, we aimed to assess the impact of different factors, including maternal HIV infection and malaria in pregnancy on the placental transfer and cord levels of IgG and IgG subclasses to a broad range of highly prevalent microbial and vaccine antigens in a sub-Saharan African country, including: *Corynebacterium diphtheriae* (*C. diphtheriae*), *Clostridium tetani* (*C. tetani*)*, Bordetella pertussis* (*B. pertussis*)*, Hib, S. pneumoniae, Shigella dysenteriae* (*S. dystenteriae*)*, Vibrio cholerae* (*V. cholerae*), hepatitis B virus (HBV), measles, RSV, *Cryptosporidium parvum* (*C. parvum*)*, Giardia intestinalis* (*G. intestinalis*), and *P. falciparum*. A better understanding of factors affecting cord IgG levels will help with designing better preventive measures and strategies for maternal and child health.

## Materials and Methods

### Study Design and Sample Collection

A total of 197 HIV-uninfected and 144 HIV-infected women were recruited among those participating in two clinical trials of antimalarial intermittent preventive treatment in pregnancy (IPTp, ClinicalTrialGov NCT00811421 for both) (Supplementary Figure 1) in the Manhiça District, Southern Mozambique (47, 48), between May 2011 and September 2012, to perform an immunology ancillary study. The first clinical trial evaluated mefloquine (MQ) as an alternative IPTp drug to sulfadoxine-pyrimethamine (SP) in HIV-uninfected pregnant women. The study arms were (1) SP, (2) single dose MQ (MQ full), and (3) split dose over 2 days MQ (MQ split). The second trial evaluated MQ as IPTp drug in HIV-infected pregnant women in whom SP is contraindicated and who received daily cotrimoxazole (CTX), and women received either three monthly doses of MQ or placebo. All women received bed nets treated with long-lasting insecticide and supplements of folic acid and ferrous sulfate. All women also received tetanus toxoid vaccination during pregnancy. At the time of the study, the intensity of malaria transmission was low/moderate (49). Antiretroviral therapy (ART) with daily monotherapy with zidovudine (AZT) was recommended when CD4^+^ T cell count was below <350 cells/μL and/or when women were in WHO HIV clinical stage III or IV (50). The number of women on ART regime was 116, of which 81 started before pregnancy and 35 at the start of the study recruitment. A total of 24 women were not in an ART regime.

At delivery, peripheral and cord blood samples from women were collected into sodium heparin and EDTA vacutainers to perform the antibody assays. Plasma samples from peripheral blood and cord blood were available for this study from 332 (195 HIV-uninfected and 137 HIV-infected) and 303 women (178 HIV-uninfected and 125 HIV-infected), respectively. There were 294 mother-cord paired samples. The extraction of the maternal blood was done before delivery, when women were admitted to the hospital.

For the detection of *P. falciparum* species, thick and thin blood smears were assessed according to standard procedures (47, 48). Fifty microliter of maternal peripheral, placental, and cord blood samples were collected on Whatman 903™ filter paper during two visits before delivery (one during second trimester and the other during third trimester) and at delivery. Real-time quantitative polymerase-chain-reaction (qPCR) assay targeting the 18S ribosomal RNA was performed (51). Tissue samples from the maternal side of the placenta (decidua) were also collected for the assessment of placental malaria. Microscopy data of peripheral and placental blood smears at delivery were available for 308 and 340 women, respectively. Peripheral and placental blood qPCR data were available for 242 and 236 women, respectively.

### Antibody Assays

Quantitative suspension array technology (qSAT) assays applying the xMAP™ technology (Luminex Corp., TX) were used to measure antigen-specific IgG, IgG1, IgG2, IgG3, and IgG4 responses to vaccine and pathogen antigens. A total of 16 recombinant proteins were selected for the analysis: diphtheria toxoid (*Corynebacterium diphtheriae*, Alpha Diagnostic DTOX15-N-500), tetanus toxin (*Clostridium tetani*, Santa Cruz SC222347), pertussis toxin (*Bordetella pertussis*, Santa Cruz SC200837), *Hib* Oligosaccharide (BEI Resources NR12268), pneumococcal surface protein A (PspA, *Streptococcus pneumoniae*, BEI Resources NR33179), shiga toxin (*Shigella dysenteriae*, BEI Resources NR4676), anti-O-specific polysaccharide (OSP, *Vibrio cholerae*, Massachusetts General Hospital, MA, USA) (52), hepatitis B surface antigen (HBsAg, Abcam ab91276), hemagglutinin (measles, Alpha Diagnostic RP655), viral protein 6 (VP6, rotavirus, Friedzgerald 80-1389), F protein (respiratory syncytial virus, BEI Resources NR31097), 17-kDA surface antigen (Cp17, *Cryptosporidium parvum*, Centers for Disease Control and Prevention, GA, USA) (53), variant-specific surface protein 5 (VSP5, *Giardia intestinalis*) (53), 42 kDA fragment of merozoite surface protein 1 (MSP1_42_, *P. falciparum*, WRAIR) (54), merozoite surface protein 2 (MSP2, *P. falciparum*, University of Edinburgh) (55), and exported protein 1 (EXP1, *P. falciparum*, Sanaria) (56). MSP1_42_ antigen was selected for representing *P. falciparum*. Eight recombinant proteins represented the most prevalent pathogens circulating in the study area (57–59) and six were from the vaccines administrated to the infants through the EPI in Mozambique (60).

qSAT assays were previously standardized and optimized to control for sources of variability (61–63). Briefly, antigens covalently coupled to MagPlex beads were added to a 96-well μClear® flat bottom plate (Greiner Bio-One) in multiplex resuspended in 50 μL of PBS, 1% BSA, 0.05% Azide pH 7.4 (PBS-BN). Fifty microliter of test samples, negative or positive controls (64) were added to multiplex wells and incubated overnight at 4°C protected from light. After incubation, plates were washed three times with PBS-Tween 20 0.05%, and 100 μL of anti-human IgG (Sigma B1140), anti-human IgG1 (Abcam ab99775), anti-human IgG2 (Invitrogen MA1-34755), anti-human IgG3 (Sigma B3523) or anti-human IgG4 (Invitrogen MA5-16716), each at their corresponding dilution, were added and incubated for 45 min. Then, plates were washed three times more and 100 μL of streptavidin-R-phycoerythrin (Sigma 42250) at the appropriate dilution were added to all wells and incubated 30 min for IgG, IgG1, and IgG3. For IgG2 and IgG4, 100 μL of anti-mouse IgG (Fc-specific)–biotin (Merck B7401) were added and incubated for 45 min, followed by another washing cycle and the incubation with streptavidin-R-phycoerythrin for 30 min. Finally, plates were washed and beads resuspended in 100 μL/well of PBS-BN. Plates were read using the Luminex 100/200 analyzer, and at least 20 microspheres per analyte were acquired per sample. Antibody levels were measured as median fluorescence intensity (MFI). Data were captured using xPonent software.

Test samples were assayed at 2 dilutions for IgG (1/250 and 1/10,000), and IgG1 and IgG3 (1/100 and1/2,500). For IgG2 and IgG4 only 1 dilution was tested (1/50) because of their usual low levels. Twelve serial dilutions (1:3, starting at 1/25) of a positive control (WHO Reference Reagent for anti-malaria *P. falciparum* human serum, NIBSC code: 10/198) were used for QA/QC and to select an optimal sample dilution for data analysis. Two blanks were also added to each plate for quality control purposes. Sample distribution across plates was designed to ensure a balanced distribution of groups. Single replicates of the assay were performed.

### Statistical Analysis

To stabilize the variance, the analysis was done on log_10_-transformed values of the MFI measurements. A positive control curve was used to select the sample dilution for each antigen-isotype/subclass-plate. The dilution nearest to the midpoint between the two positive control curve serial dilutions ranging the maximum slope was chosen. Plates were normalized using the positive control curve in each plate and the average positive control curve from all plates. The MFI values of samples were multiplied by the corresponding normalization factor (MFI value of the chosen dilution from the average positive control curve divided by the MFI value of same dilution in the plate curve).

The Shapiro-Wilk test of normality confirmed that most of the antibody data were not normally distributed. The Chi-square and the non-parametric Wilcoxon-Mann-Whitney tests were used to compare categorical and continuous variables, respectively, between HIV-infected and HIV-uninfected women. Comparisons of crude Ig levels across antigens and subclasses between HIV exposure groups were assessed by Wilcoxon-Mann-Whitney tests, and between ART groups, were assessed by Kruskal-Wallis and Dunn's tests. Kruskal-Wallis and Dunn's tests were also performed in order to compare IgG subclass placental transfer for each antigen, represented as the cord/mother ratios. Univariable linear regression models were fit to determine the effect of variables on the cord blood antibody levels (log_10_) or the cord blood/mother ratio (log_10_) (Supplementary Material 1). The variables considered in this analysis were log_10_ maternal antibody levels, maternal HIV infection, *P. falciparum* exposure, PM (acute, defined by the presence of parasites on sections without malaria pigment; chronic, by presence of parasites and pigment; or past, by the presence of pigment alone), age, gravidity (defined as *primigravidae* and *multigravidae*), maternal anemia (defined as hemoglobin level <11 g/dL), low birth weight (defined as <2,500 g at birth), prematurity (defined as delivery before 37 weeks of gestational age), gestational age [measured by Ballard score (65)], treatment (defined as MQ or placebo in the HIV-infected women ancillary study and MQ full, MQ split or SP in HIV-uninfected women ancillary study), ART received before pregnancy or at recruitment, CD4^+^ T cell counts (<350 or ≥350 cells/μL), HIV viral load (<400, 400–999, 1,000–9,999, and >9,999 copies/mL), and seasonality (dry or rainy). Exposure to *P. falciparum* was computed as the sum of the maternal IgG antibody levels (MFI) for the following immunogenic *P. falciparum* antigens: MSP1_42_, MSP2, and EXP1, as antibody levels to these antigens have been shown to reflect exposure to malaria (66, 67). Seasonality was computed for each woman based on the pregnancy period—if at least four of the pregnancy months fell under the category of rainy period (November through April), the season was defined as such. In any other case, the season was defined as dry. A base multivariable model including maternal antibody levels, maternal HIV infection and *P. falciparum* exposure was established for each antigen and IgG or IgG subclass. Base model for MSP1_42_ did not include *P. falciparum* exposure as this variable includes antibodies to this antigen. We performed additional regression models testing exhaustively all possible combinations of predictor variables (added to our base model) and selected the models based on the Akaike information criterion (AIC), Bayesian information criterion (BIC) and adjusted r-square parameters. All *p*-values were considered statistically significant when <0.05 after adjusting for multiple testing through Benjamini-Hochberg. All data collected were pre-processed, managed, and analyzed using the R software version 3.6.3 and its package *devtools* (68). The *ggplot2* package was used to perform boxplot graphs (69). The *FactoMineR* and *factoextra* packages were used to perform Principal Component Analysis (PCA) (70, 71).

## Results

### Description of Participants

A total of 341 women (197 HIV-uninfected and 144 HIV-infected) participated in the study (Table 1). HIV-infected women were older than the HIV-uninfected and there were more *primigravidae* among the HIV-uninfected. HIV-infected women had significantly more anemia than the HIV-uninfected. There were no significant differences in birth weight or prematurity between infants born to HIV-infected and those born to HIV-uninfected women. Among the 155 infants born from HIV-infected women, eight tested HIV-positive at 6 weeks of age by polymerase chain reaction (PCR) analysis performed following national guidelines. Placental histology was performed on 307 samples from study participants, of which three had acute PM and eight past PM. In total, 20 women had PM (positive in placental blood, by microscopy or PCR at delivery, or acute or past PM by histology), but there were no differences by HIV infection. Peripheral malaria (positive in peripheral blood by microscopy or PCR at any of the visits during pregnancy) was detected in 51 women, but there were no differences by HIV infection. Finally, *P. falciparum* exposure was lower among HIV-infected women.

**Table 1:** Characteristics of study participants.

	**All *N* = 341**	**HIV-uninfected *N* = 197**	**HIV-infected *N* = 144**	***p*-value^**a**^**
Age^a^ [years median (IQR)]	25.0 [19.0; 29.0]	21.0 [18.0; 28.0]	27.0 [22.0; 31.0]	<0.001
Gravidity (*n*, %)				<0.001
*Multigravidae*	259 (76.0)	128 (65.0)	131 (91.0)	
*Primigravidae*	82 (24.0)	69 (35.0)	13 (9.0)	
Maternal hemoglobin (*n*, %)				0.025
Anemia (<11 g/dL)	208 (61.5)	109 (56.2)	99 (68.8)	
Normal (≥11 g/dL)	130 (38.5)	85 (43.8)	45 (31.2)	
Birth weight (*n*, %)				NS
Low (<2,500 g)	29 (8.5)	17 (8.6)	12 (8.33)	
Normal (≥2,500 g)	312 (91.5)	180 (91.4)	132 (91.7)	
Prematurity (*n*, %)				NS
No (≥37 weeks)	312 (94.3)	181 (95.3)	131 (92.9)	
Yes (<37 weeks)	19 (5.7)	9 (4.7)	10 (7.1)	
Treatment				<0.001
MQ	71 (20.9)	0 (0.0)	71 (49.7)	
MQ full	68 (20.8)	68 (34.5)	0 (0.0)	
MQ split	73 (21.5)	73 (37.1)	0 (0.0)	
Placebo	72 (21.2)	0 (0.0)	72 (50.3)	
SP	56 (16.5)	56 (28.4)	0 (0.0)	
ART (*n*, %)				NP
No	24 (7.1)	–	24 (17.1)	
Yes	116 (34.4)	–	116 (82.9)	
CD4^+^ T cell counts (*n*, %)				NP
Lower (<350 c/μL)	40 (12.3)	–	40 (31.2)	
Higher (≥350 c/μL)	88 (27.1)	–	88 (68.8)	
HIV viral load (copies/mL)				NP
<400	21 (6.4)	–	21 (16.0)	
(400–999)	41 (12.5)	–	41 (31.3)	
(1,000–9,999)	48 (14.6)	–	48 (36.6)	
>9,999	21 (6.4)	–	21 (16.0)	
Placental malaria^b^ (*n*, %)				NS
No	321 (94.1)	184 (93.4)	137 (95.1)	
Yes	20 (5.9)	13 (6.6)	7 (4.9)	
Peripheral malaria^c^ (*n*, %)				NS
No	290 (85.0)	165 (83.8)	125 (86.8)	
Yes	51 (15.0)	32 (16.2)	19 (13.2)	
*P. falciparum* exposure (log_10_ MFI IgG)	5.27 [5.19; 5.34]	5.29 [5.21; 5.35]	5.26 [5.18; 5.33]	0.011

*For numerical variables, the median and first and third quartile, in brackets, are given. For the categorical variables, the number of individuals for each group and percentages, in parentheses, are given*.

a *For the age, the Wilcoxon-Mann-Whitney test was used to compare differences between median values. For the categorical variables, the Chi-square test was used*.

b *Placental malaria was considered positive if there was any evidence of P. falciparum placental parasitemia by any method*.

c *Peripheral malaria was considered positive if there was any evidence of P. falciparum peripheral parasitemia by any method*.

*The statistical significance was considered when p < 0.05; MQ, mefloquine; NS, not significant; NP, not-performed tests; SP, sulfadoxine-pyrimethamine*.

### Profile of Antibody Levels in Cord Blood

**Figure 1:**
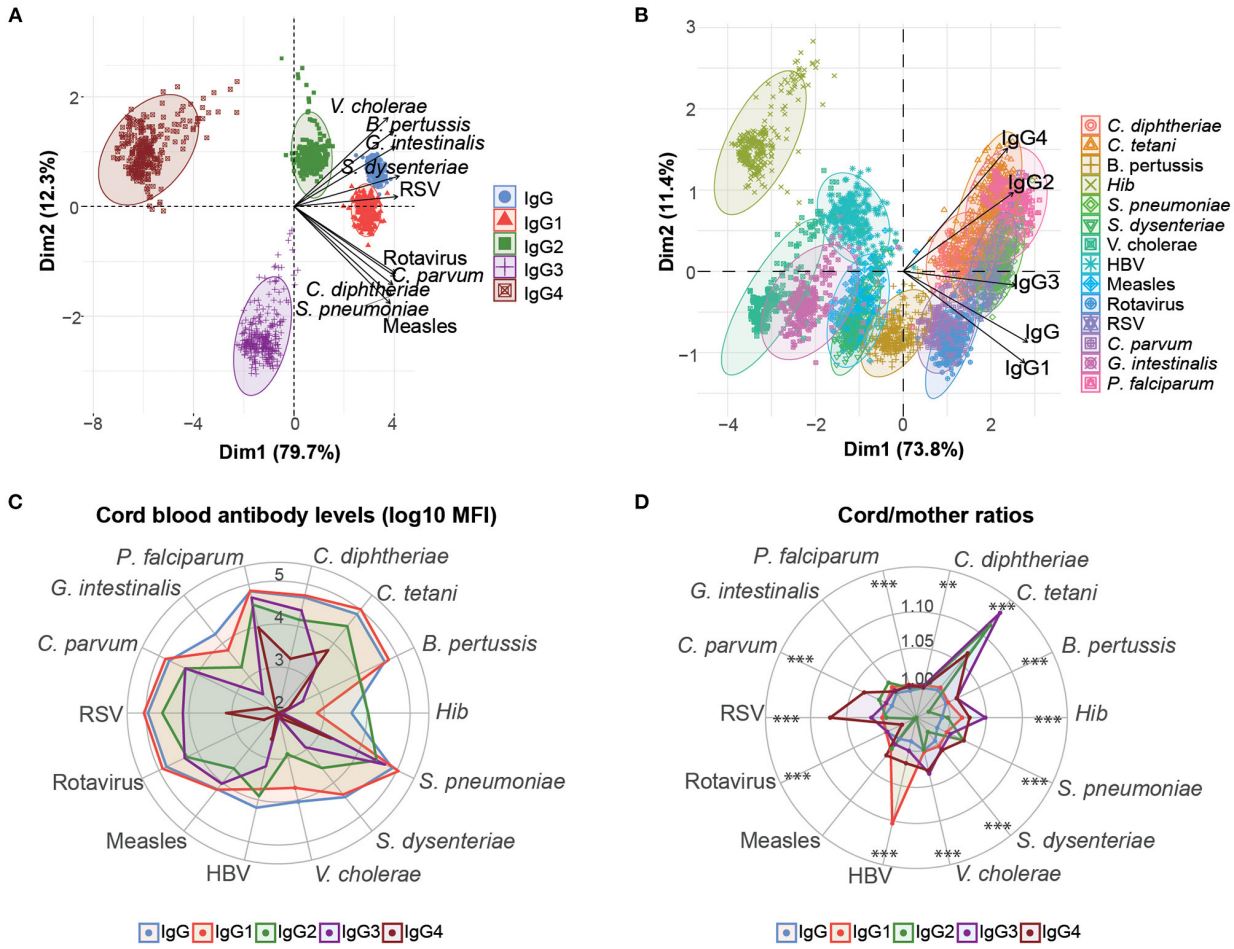
Overview of cord blood levels of IgG and IgG subclasses for all women. Principal component analysis (PCA) plots of cord IgG and IgG subclass levels against all antigens clustered by subclass type **(A)** and antigen type **(B)**. The two principal components (Dim 1, Dim2) that explained the highest percentage of the variance of the data (percentage in parenthesis) were chosen for representation. The arrows in **(A)** and **(B)** represent how the variables contribute to each of the two principal components. **(C)** Medians of IgG and IgG subclass levels (log_10_ MFI) in cord blood for each antigen. **(D)** Medians of IgG and IgG subclass placental transfer for each antigen, represented as the cord/mother ratios. Medians of IgG subclass placental transfer for each antigen were compared by Kruskal-Wallis test and *p*-values were adjusted for multiple testing by the Benjamini-Hochberg approach (False Discovery Rate 5%). ***p < 0.001, **p < 0.01.

PCA of the cord antibody levels and maternal antibody levels separately, including IgG, IgG1, IgG2, IgG3, and IgG4 to all antigens tested, were performed to reduce the dimensionality of the data and get insights into the overall antibody patterns. Cord and maternal PCA looked very similar (data not shown). Cord antibody responses clearly clustered by IgG subclasses (Figure 1A). The distribution of the IgG subclass clusters across the PCA dimensions was influenced by the antigens. Specifically, IgG and IgG1 clusters were closer, showing similar responses, whereas IgG4 and IgG3 were the most distant. Cord antibody responses also clustered by antigens (Figure 1B). *Hib* cluster was clearly separated from the rest, indicating a different antibody profile (Figure 1B). Overall, PCA results suggested different antibody profiles depending on the antigen specificity. Consistently, median IgG and IgG1 levels were higher than the rest of IgG subclasses and were both similar between them for most of the antigens with the exception of *Hib* (Figure 1C). IgG2 had lower median levels than IgG1, followed by IgG3 and the lowest levels were shown for IgG4.

We also determined the placental transfer of antibodies, measured as the ratio of cord blood levels to the maternal levels. The efficiency of placental transfer was different depending on the IgG subclass in all antigens with the exception of measles and *G. intestinalis* (Figure 1D). The transfer efficiency was greatest for IgG1, IgG3, or IgG4 depending on the antigen. IgG1 placental transfer was significantly higher for *C. diphtheriae, P. falciparum*, HBV, and rotavirus. IgG3 placental transfer was significantly higher for *B. pertussis, C. tetani, Hib*, and *V. cholerae*. IgG4 placental transfer was significantly greater for *C. parvum, S. dysenteriae*, and RSV. The least efficiently transferred subclass was IgG2 for most of the antigens, with the exception of *S. pneumoniae* for which IgG2 and IgG4 were the greatest.

### Altered Maternal and Cord Blood Antibody Levels in HIV-Infected Women

**Figure 2:**
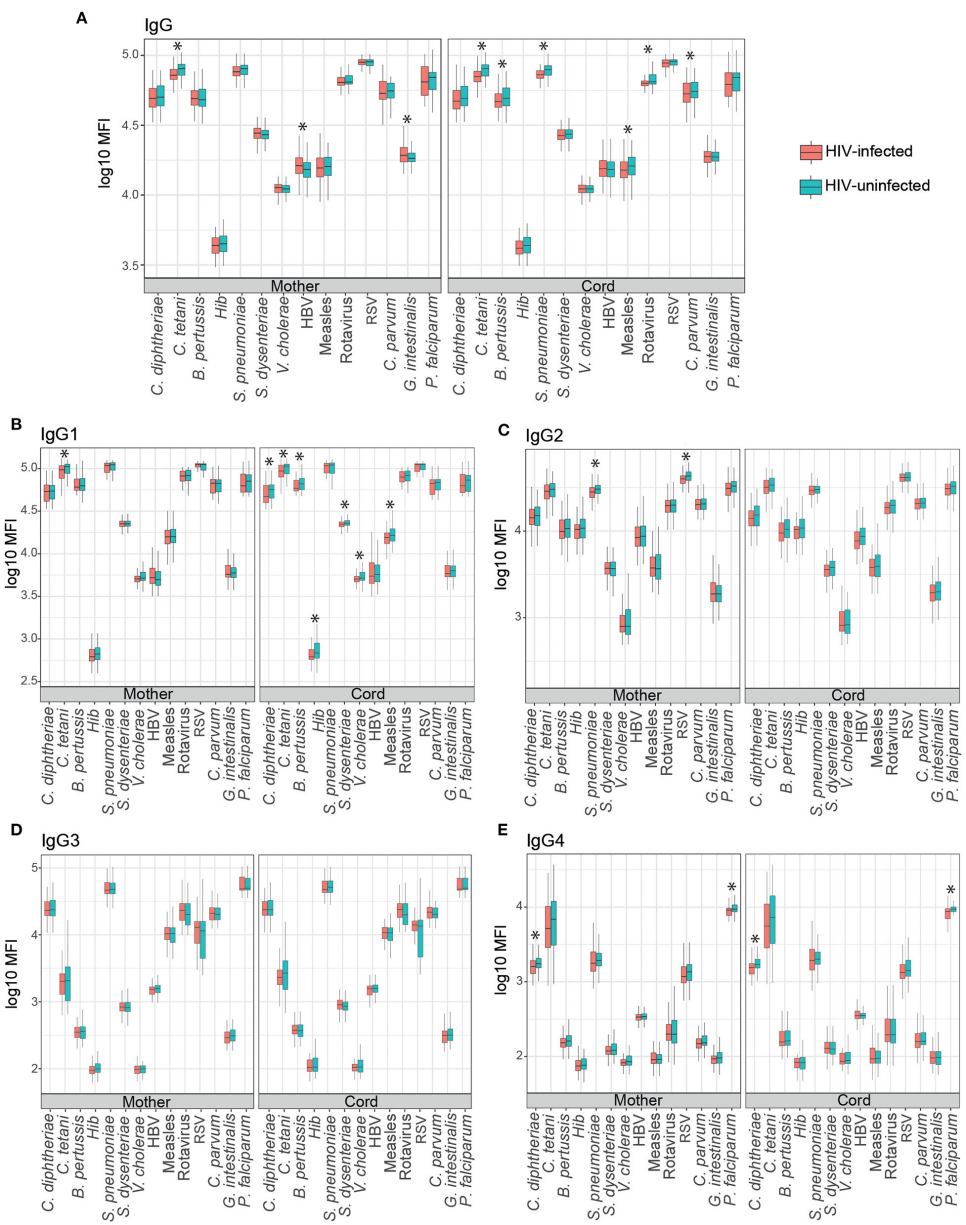
Mother and cord blood antibody levels (log_10_ MFI) in HIV-positive and HIV-negative women. Boxplots illustrate the medians and the interquartile range for IgG **(A)**, IgG1 **(B)**, IgG2 **(C)**, IgG3 **(D)**, and IgG4 **(E)** subclasses in HIV-positive (red) and HIV-negative women (blue). Levels between groups were compared by Wilcoxon-Mann–Whitney test and *p*-values were adjusted for multiple testing by the Benjamini-Hochberg approach (False Discovery Rate 5%). Statistically significant differences are highlighted with an asterisk.

We first compared total IgG levels and IgG subclasses in maternal and cord blood from HIV-infected and HIV-uninfected women. Significant differences in maternal antibody levels were only detected for *C. tetani* (IgG and IgG1), *S. pneunomiae* and RSV (IgG2), and *C. diphtheriae* and *P. falciparum* (IgG4), with lower antibody levels in HIV-infected compared to HIV-uninfected women (Figure 2). In contrast, higher *G. intestinalis* and HBV IgG levels were found in HIV-infected women (Figure 2A).

Total IgG cord blood levels were lower in HIV-infected than HIV-uninfected women for *C. tetani, B. pertussis, S. pneumoniae*, measles, rotavirus, and *C. parvum*. Similarly, HIV-infected women had lower IgG1 cord levels for *C. diphtheriae, C. tetani, B. pertussis, Hib, S. dysenteriae, V. cholerae*, and measles for IgG1 (Figure 2B). No differences were observed between groups for IgG2 and IgG3 (Figures 2C,D), whereas lower *C. diphtheriae and P. falciparum* IgG4 levels were also found in cord blood of HIV-infected than HIV-uninfected women (Figure 2E).

We also compared cord blood levels of IgG and IgG subclasses between HIV-infected women taking ART before pregnancy, at recruitment or without ART regime during pregnancy. The only significant differences were observed for *S. dysenteriae* and RSV IgG4 (Supplementary Figure 2E). HIV-infected women taking ART before pregnancy had higher cord blood levels of IgG4 against S. *dysenteriae* and RSV than those women taking no ART (*p* < 0.05 in Dunn's test adjusted by Benjamini-Hochberg, False Discovery Rate 5%), but no differences were found among women taking ART at recruitment than those not taking ART. Similarly, no differences were found between women taking ART before pregnancy or at recruitment.

**Figure 3:**
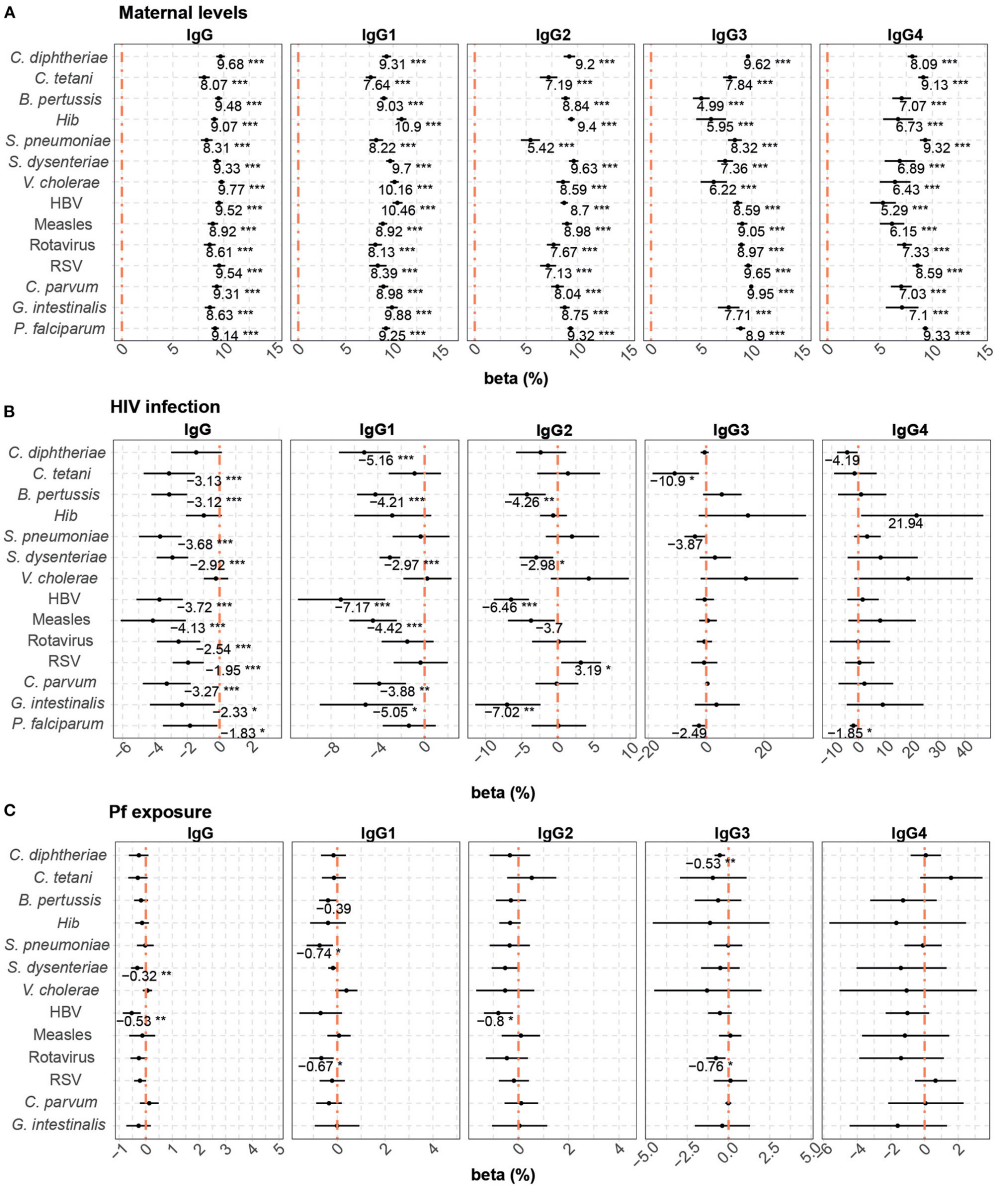
Difference of IgG and IgG subclass levels in cord blood by study factors. Forest plots show the effect (in percentage) of maternal antibody levels **(A)**, HIV infection **(B)** and *P. falciparum* exposure (Pf exposure) **(C)** on cord blood levels of IgG and IgG subclasses for all the antigens tested. The differences in percentage correspond to beta-transformed values (%) calculated from the beta values obtained in the multivariable models. Beta transformed values (%) are displayed when raw *p*-values are significant. Asterisks are shown when adjusted *p*-values by Benjamini-Hochberg are significant (False Discovery Rate 5%). ***p < 0.001, **p < 0.01, *p < 0.05.

Multivariable models were also performed to assess the factors associated with cord blood levels of total IgG and IgG subclasses. Maternal antibodies, HIV infection and *P. falciparum* exposure were included in the models. Maternal antibody levels had the strongest positive correlation with cord antibody levels for all the antigens and subclasses (Figure 3A). However, the effect of maternal antibody levels was more variable for IgG3-4 than for total IgG and IgG1 subclass. A 10% increase in maternal total IgG levels was associated with increases from 8.1 to 9.7% in total IgG cord blood levels, depending on the antigen. For IgG subclasses, a 10% increase in maternal antibody levels was associated with increases of cord blood levels from 7.6 to 10.9% for IgG1, 5.4 to 9.6% for IgG2, 5 to 9.9% for IgG3, and 5.3 to 9.3% for IgG4.

Maternal HIV infection (Figure 3B) had a negative effect on total IgG cord blood levels to all antigens, except for *C. diphtheriae, Hib*, and *V. cholerae*. HIV infection was associated with a reduction ranging from 1.83 to 4.13% in the IgG cord blood levels. For IgG1, HIV infection negatively impacted cord blood levels against *C. diphtheriae, B. pertussis, S. dysenteriae*, HBV, measles, *C. parvum*, and *G. intestinalis* (from 2.97 to 7.17% reduction). For IgG2, a reduction was observed against *B. pertussis, S. dysenteriae*, HBV, and *G. intestinalis* (from 2.98 to 7.02% reduction), whereas HIV was associated with an increase of 3.19% of IgG2 to RSV. Finally, we only detected a negative effect of HIV infection on IgG3 levels to *C. tetani* (10.90% reduction) and IgG4 to *P. falciparum* (1.85% reduction).

*P. falciparum* exposure was negatively associated with cord blood IgG levels against *S. dysenteriae* and HBV, IgG1 against *S. pneumoniae* and rotavirus, IgG2 against HBV and IgG3 against *C. diphtheriae* and rotavirus (Figure 3C). Depending on the IgG sublcass and antigen, 10% increase in *P. falciparum* exposure reduced the cord blood levels ranging from 0.32 to 0.80%.

Previous studies suggest that PM rather than peripheral malaria may affect cord blood levels and transplacental transfer of antibodies and lead to adverse outcomes due to the damaged placental tissue (37, 72, 73). Therefore, we explored the effect of PM on cord blood levels in multivariable models without *P. falciparum* exposure despite the low number of women with any evidence of PM. PM had no significant associations with antibody cord blood levels (data not shown). When analyzing HIV-infected women only, PM was associated with lower *B. pertussis* IgG1, *C. diphtheriae* IgG2 and HBV IgG3 levels in cord blood (Supplementary Figure 3).

**Figure 4:**
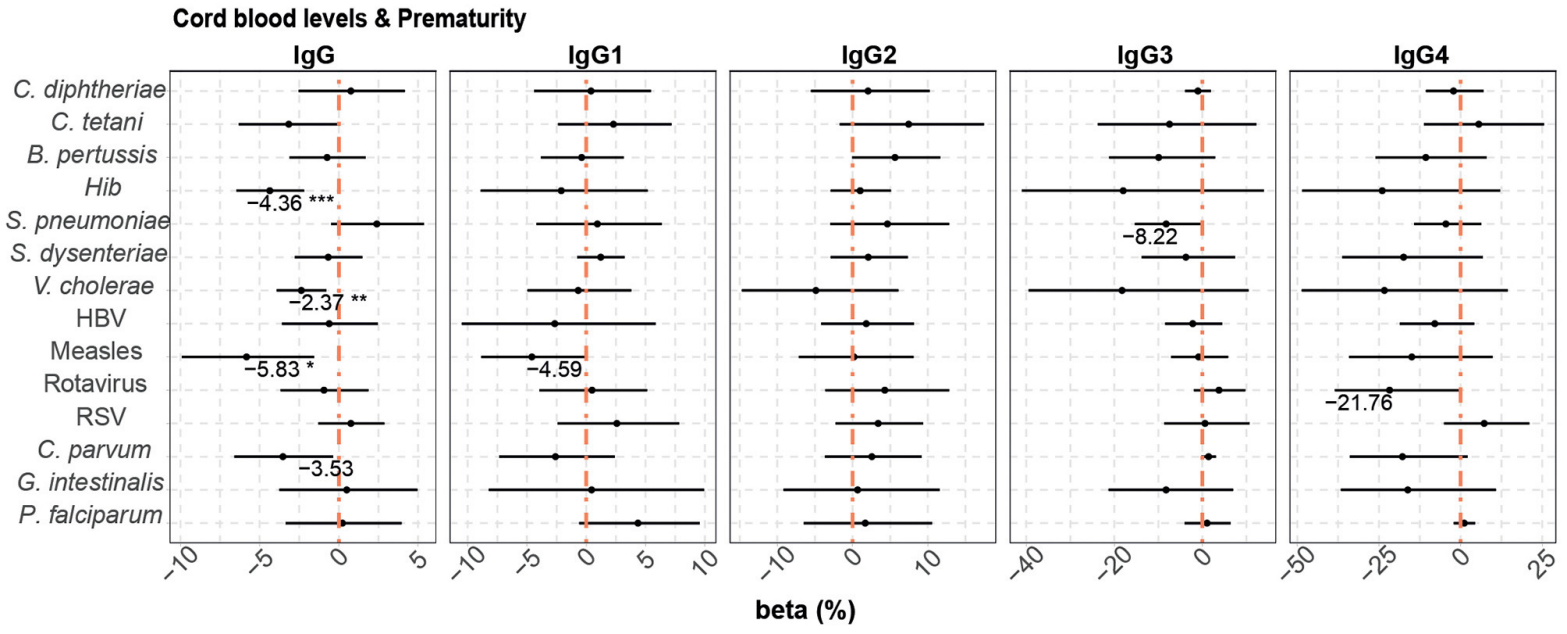
Difference of IgG and IgG subclass levels in cord blood by prematurity. Forest plots show the effect (in percentage) of prematurity on cord blood levels of IgG and IgG subclasses, for all the antigens tested. The differences in percentage correspond to beta-transformed values (%) that were calculated from the beta values in the multivariable models. This beta value was obtained when prematurity was included in multivariable models with maternal antibody levels, HIV infection and *P. falciparum* exposure. Beta transformed values (%) are displayed when raw *p*-values are significant. Asterisks are shown when adjusted *p*-values by Benjamini-Hochberg are significant (False Discovery Rate 5%). ***p < 0.001, **p < 0.01, *p < 0.05.

Prematurity, previously shown to have a detrimental effect on placental transfer of antibodies (74), was added to the multivariable model, which included the variables maternal antibody levels, HIV infection and *P. falciparum* exposure, as it increased the quality (AIC) of some of the models. Prematurity (Figure 4) was associated with lower cord blood total IgG levels against *Hib* (4.36% reduction compared with term cord blood levels)*, V. cholerae* (2.37% reduction), measles (5.83% reduction), and *C. parvum* (3.53% reduction without statistical significance after adjusting for multiple testing).

The rest of the variables (age, maternal anemia, gravidity, low birth weight, IPTp treatment, seasonality; and CD4^+^ T cell counts, ART and viral load for HIV-infected women) did not provide any added value to the multivariable models and were not included.

### Altered Placental Transfer of Antibodies in HIV-Infected Women

**Figure 5:**
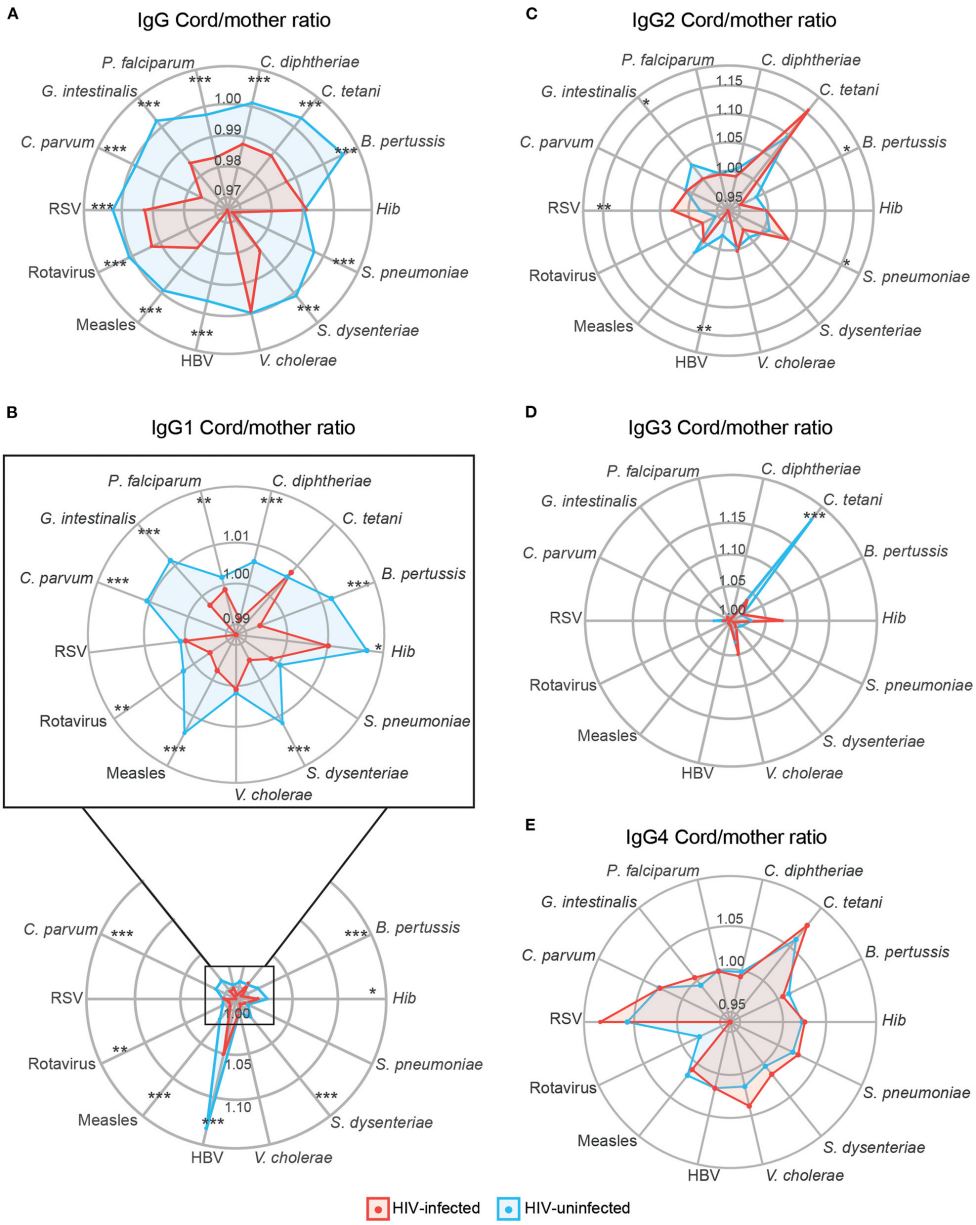
Antibody placental transfer in HIV-positive and HIV-negative women. Radar charts representing the medians of each antigen antibody cord/mother ratio in HIV-positive and HIV-negative women for IgG **(A)**, IgG1 subclass **(B)**, IgG2 **(C)**, IgG3 **(D)**, and IgG4 **(E)**. Ratios between HIV-positive and negative women were compared by Wilcoxon-Mann-Whitney test and *p*-values were adjusted for multiple testing by the Benjamini-Hochberg approach (False Discovery Rate 5%). Statistically significant differences between HIV-positive and negative women ratios are highlighted with an asterisk. ***p < 0.001, **p < 0.01, *p < 0.05. HIV-positive women are represented in red and HIV-negative women in blue.

Placental transfer of total IgG and IgG1 was significantly lower in HIV-infected women for all antigens except for *Hib* and *V. cholerae* (total IgG) and *C. tetani, S. pneumoniae, V. cholera*, and RSV (IgG1) (Figures 5A,B). For IgG2, only *G. intestinalis, B. pertussis*, and HBV had significantly lower transfer in HIV-infected women, while *S. pneumoniae* and RSV had higher transfer in HIV-infected women (Figure 5C). For IgG3, only *C. tetani* had a significantly lower transfer in HIV-infected compared to HIV-uninfected women (Figure 5D). No significant differences in placental transfer between the two groups were found for IgG4 (Figure 5E).

We compared placental transfer of IgG and IgG subclasses between HIV-infected women taking ART before pregnancy, at recruitment or without ART regime. No significant differences were found, with the exception of *C. parvum* IgG4 (Supplementary Figure 4E), for which HIV-infected women taking ART before pregnancy or at recruitment had higher placental transfer than those not taking ART (*p* < 0.05 in Dunn's test adjusted by Benjamini-Hochberg, False Discovery Rate 5%). There were no differences between taking ART before pregnancy or at recruitment in the *C. parvum* IgG4 placental transfer.

**Figure 6:**
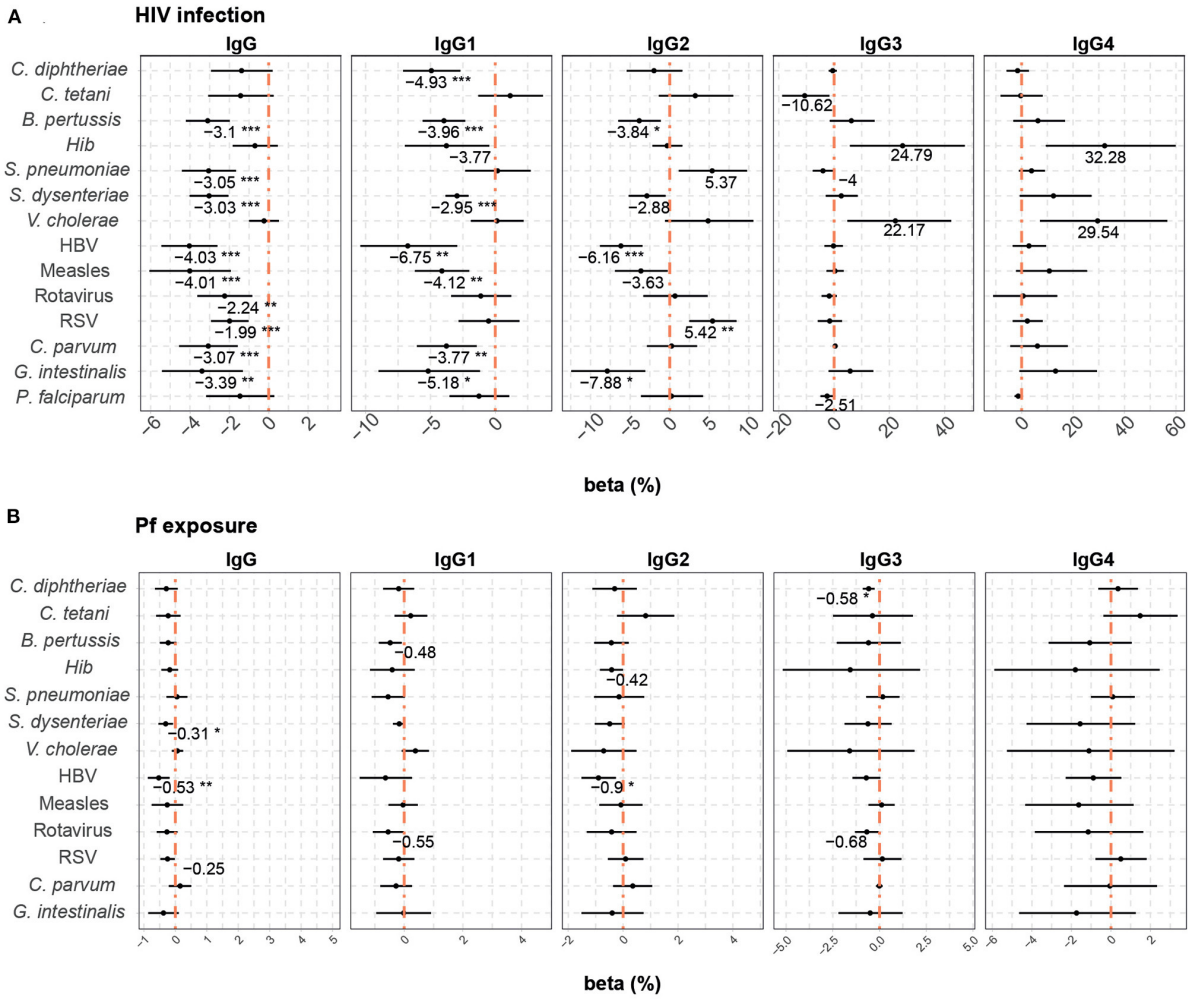
Difference of IgG and IgG subclass placental transfer by study factors. Forest plots show the effect (in percentage) of HIV infection **(A)** and *P. falciparum* exposure (Pf exposure) **(B)** on transplacental transfer of IgG and IgG subclasses for all the antigens tested. The differences in percentage correspond to beta-transformed values (%) that were calculated from the beta values obtained in the multivariable models. Beta transformed values (%) are displayed when raw *p*-values are significant. Asterisks are shown when adjusted *p*-values by Benjamini-Hochberg are significant (False Discovery Rate 5%). ***p < 0.001, **p < 0.01, *p < 0.05.

In multivariable models including HIV infection and *P. falciparum* exposure, HIV infection (Figure 6A) in general was associated with reduced placental transfer of total IgG and IgG1 (from 1.99 to 6.75% reduction depending on the antigen). HIV infection was also associated with a reduced transfer of IgG2 against *B. pertussis*, HBV, and *G. intestinalis*, but an increase in IgG2 RSV transfer (5.42% increase). Although adjusted *p*-values were not significant, a similar trend of positive correlation was found for *S. pneumoniae* IgG2 and *Hib* and *V. cholerae* IgG3 and IgG4.

*P. falciparum* exposure (Figure 6B) had a negative effect on the placental transfer of antibodies for some antigens. An increase of 10% in *P. falciparum* exposure reduced the placental transfer of total IgG against *S. dysenteriae* and HBV by 0.31 and 0.53%, respectively, IgG2 against HBV by 0.90% and IgG3 against *C. diphtheriae* by 0.58%.

PM, in contrast to *P. falciparum* exposure, did not have any impact on transplacental transfer of antibodies in exploratory analyses and did not improve any of the models, although it had similar correlation trends for total IgG. Nevertheless, PM was associated with a diminished placental transfer of IgG1 *B. pertussis* in HIV-infected women (Supplementary Figure 5).

**Figure 7:**
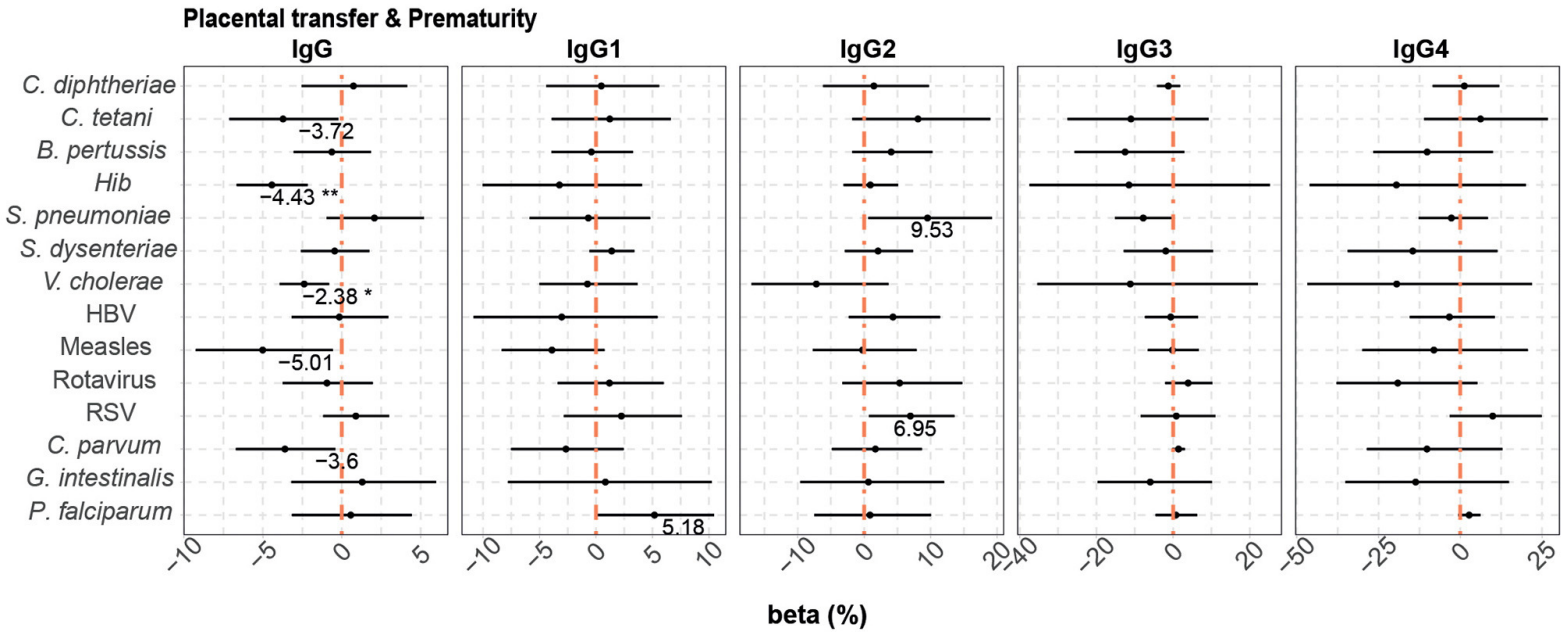
Difference of IgG and IgG subclass placental transfer by prematurity. Forest plots show the effect (in percentage) of prematurity on transplacental transfer of IgG and IgG subclasses, for all the antigens tested. The differences in percentage correspond to beta transformed values (%) that were calculated from the beta values in the multivariable models. This beta value was obtained when prematurity was included in multivariable models with HIV infection and *P. falciparum* exposure. Beta transformed values (%) are displayed when raw *p*-values are significant. Asterisks are shown when adjusted *p*-values by Benjamini-Hochberg are significant (False Discovery Rate 5%). **p < 0.01, *p < 0.05.

When prematurity was added to the multivariable models that included HIV infection and *P. falciparum* exposure, this additional covariable had a negative effect on placental transfer of only *Hib* and *V. cholerae* total IgG antibodies (4.43 and 2.38% reduction in premature vs. term newborns, respectively) (Figure 7).

No additional variables were included in the multivariable analysis as they did not provide any added value to the model.

## Discussion

Our comprehensive analysis of maternal and cord plasma total IgG and IgG subclasses against a wide range of microbial and vaccine antigens allowed a deep immunoprofiling, that is essential to decipher the mechanisms affecting antibody placental transfer and maternal and newborn immunity in women chronically exposed to pathogens. We confirmed that the main determinant of cord total IgG and IgG subclass levels are the corresponding maternal antibody levels, and that maternal HIV infection is associated with a reduction of total IgG levels in the cord due to low maternal levels, but also to a reduction of IgG and IgG1 placental transfer.

Maternal and cord blood antibody levels are usually correlated in many studies, suggesting that maternal levels are the main determinant for transfer efficiency (14, 75, 76). However, the effect of HIV infection on placental transfer is not consistent among studies and prior analyses of the effect of HIV on maternal and cord blood levels have mainly focused on total IgG (16, 28, 29, 33, 39, 41–43, 46). Our results showed that HIV infection reduced the total IgG maternal levels for some antigens, the cord blood levels overall, and also had a negative effect on transplacental transfer of total IgG antibodies. It is interesting that although we found higher maternal HBV and *G. intestinalis* IgG levels among HIV-infected women, cord blood levels and transplacental transfer were lower than in HIV-uninfected women. Higher maternal antibody levels against these pathogens in HIV-infected women may be due to an increased susceptibility to co-infections with these pathogens, as described before (77–79), but it seems that they are not being transferred as efficiently as in HIV-uninfected women. This could be due to hypergammaglobulinemia, demonstrated to be common among HIV-infected individuals (80) and previously shown to impair transplacental transfer of antibodies (14, 16, 35), or due to an impairment of the Fc receptors caused by maternal HIV infection (16).

Our results are consistent with previous studies reporting that HIV infection led to a reduction of the cord blood levels and transplacental transfer of total IgG against *B. pertussis* (29, 42)*, C. tetani* (28, 29, 40, 42)*, S. pneumoniae* (33, 40, 42, 44, 81), RSV (82, 83), and measles (39, 40). Some studies also found a negative effect on *Hib* (29, 81) that is not appreciated in our study, although we found reduced IgG1 levels in cord in univariable analyses. However, our results differ from other studies that did not find any effect of HIV status on IgG levels against *C. diphtheriae* (38)*, C. tetani* (33, 38), *S. pneumoniae* (46, 83), HBV (38), and measles (33, 38). These discrepancies could be due to the different geographical areas, statistical methods used, small sample sizes in the previous studies, type of antigen tested and the serological method applied.

IgG subclasses may be elicited differently depending on the pathogen, the antigen or the epitope (84), and the differences in Fc region between IgG subclasses, which mediates effector functions, confers them different roles during infection and pathogen clearance (84–86). The efficiency of the antibody placental transfer varies for each subclass due to differential affinity of the receptors FcRn (19). We found that the efficacy of IgG placental transfer also depended on the antigen. IgG1, IgG3, or IgG4 transferred better than IgG2, except for *S. pneumoniae*, for which IgG2 transfer was significantly higher. This was unexpected as classically it has been described that the greatest transport occurs for IgG1, followed by IgG4, IgG3, and finally IgG2 (20), although a recent report showed a different hierarchy of subclass transfer and identified a number of other studies that also observed different transfer efficiencies, depending on the antigen tested (21). However, IgG1 levels were the highest for almost all antigens in cord blood, probably because of the overall higher levels of this IgG subclass in maternal blood. One exception was *Hib* that presented higher IgG2 cord levels than IgG1, although IgG1 transplacental transfer was higher than IgG2 consistently with previous studies (87). The mothers had an IgG2-predominant response to *Hib*, and consequently higher IgG2 than IgG1 levels were found in cord blood as previously described (88, 89).

In our multivariable models, we observed that HIV infection reduced mainly IgG1 cord levels due to a diminished transplacental transfer, similarly to IgG. Interestingly, maternal HIV infection increased the placental transfer of IgG2 to RSV and had a positive effect on RSV IgG2 cord blood levels, although IgG2 maternal levels were lower in HIV-infected women. To our knowledge, an increased placental transfer by HIV infection has not been described before. This may have implications for maternal immunization with RSV vaccines under development. Natural RSV infection seems to elicit an age-dependent IgG1, IgG2, and IgG3 response against the F protein, the major target of the host's immune response (90, 91), and of some RSV vaccines (92). Antibodies binding to the F protein are protective (93), and IgG1 is the IgG subclass mainly produced in response to RSV infection (94, 95) and an IgG1 monoclonal antibody against RSV F protein with neutralizing function, has shown to be effective (96), suggesting that the protective response is predominantly IgG1. Here, we found that total IgG and IgG1 against RSV F protein had the highest levels in cord blood compared to other subclasses, but HIV infection reduced total IgG cord blood levels and placental transfer. Therefore, HIV infection could compromise the levels of RSV neutralizing antibodies transferred to the newborn and, consequently, diminish the effectivity of an RSV vaccine.

Regarding other variables, our data did not show any significant association between CD4^+^ T cell counts or HIV viral load on cord blood levels and transplacental transfer of antibodies. Even though these results agree with previous studies that did not find any associations (29, 81, 97), other reports describe that lower CD4^+^ T cell counts and higher HIV viral load lead to a reduction of the transfer of some pathogen-specific antibodies and vaccines such as measles and *S. pneumoniae* (37, 43, 98). Some studies describe that HIV-infected women receiving ART transferred higher pathogen-specific antibodies than those who were not under ART (97) or who initiated it during pregnancy (99). However, in our cohort we did not find any significant associations in regards to ART. The discrepancies between studies could be explained by the different ART drug regime type.

At the time of the study, malaria transmission intensity was very low in the area and only a few women had active malaria during pregnancy. Nonetheless, we found a negative correlation between *P. falciparum* exposure and both placental transfer and cord blood antibody levels for some antigens and IgG subclasses. Previous studies are contradictory, as some found that PM led to a reduction of the transplacental transfer of some pathogen-specific IgG to *C. tetani* (34), measles (35, 39), RSV (37) and *S. pneumoniae* (33), but others did not find any effect for IgG against *C. diphtheriae* (37, 38), *C. tetani* (28, 35, 38)*, Hib* (37), HBV (38), measles (38) RSV (36), and *S. pneumoniae* (37). Discrepancies between studies could be due to differences in study sites, prevalence of malaria, sample sizes, type of antigens, sensitivities of serological methods, exposure to the pathogens tested, and other co-infections. We also found that *P. falciparum* exposure was lower among HIV-infected women. Although all HIV-infected women were on prophylactic treatment with CTX, a broad-spectrum antibiotic that has been suggested to be protective against malaria (100, 101), HIV-negative women were also on IPTp with SP or MQ that has been demonstrated to be effective (47). Thus, this lower exposure could be more related to the fact that exposure to *P. falciparum* was computed as the sum of the maternal IgG antibody levels against three immunogenic *P. falciparum* antigens, and maternal HIV infection could reduce the IgG levels against them.

We found prematurity to be associated with lower cord blood IgG levels and placental transfer for some antigens, as previous studies have shown (74, 102, 103), although the effect was not consistent among subclasses. It has already been reported that the greatest transport occurs in the third trimester of gestation (17), and due to this fact, preterm infants may have lower amounts of transplacental IgG than term infants (74, 103, 104).

Our results are important for maternal immunization implementation in settings with a high prevalence of HIV infection. Here, the only vaccine given during pregnancy was tetanus. Although HIV infection was associated with lower maternal and cord blood tetanus toxoid IgG and IgG1 levels in univariable models, HIV did not affect cord blood IgG1 levels in multivariable models adjusted by maternal levels. Systemic tetanus vaccination during pregnancy has been implemented in Africa and has demonstrated a high efficacy (105). Pertussis vaccination in pregnancy has also been implemented in some countries such as Canada, US, and UK (106, 107), but not in Africa. Acellular pertussis vaccine induces mainly IgG and IgG1 responses that are thought to confer protection (108–110). We found lower cord blood levels and a reduced placental transfer of IgG and IgG1 against *B. pertussis* among HIV-infected women and those exposed to *P. falciparum*. These results highlight the need for further studies assessing the impact of these infections on pertussis vaccine efficacy and antibody placental transfer when implemented in pregnant women from African countries.

As study limitations, we could not establish the threshold of protection of antibody levels, therefore it is difficult to infer the clinical relevance of the reductions in antibodies detected in cord blood from the HIV-infected women. We could not measure hypergammaglobulinemia, which has been associated with a reduced transplacental transfer of antibodies (35–37), and demonstrated to be induced by chronic infections such as HIV, malaria or helminthiasis (80, 111, 112). Also, diabetes may have an impact on placental transfer of antibodies (31), but this diagnosis was not available in our cohort. Another limitation is that this study was only performed in a Mozambican cohort, therefore results obtained may not be representative of other populations. Specifically, IgG allotype diversity, or ethnic/geographic differences in FcRn, may impact the effect of HIV and malaria on placental transfer. As HIV-infected mothers also receive CTX, it could potentially limit antigen exposure and, therefore, antibody responses. Finally, we had to assess malaria exposure instead of PM due to the low number of PM cases and consequently our results are not comparable with those assessing PM.

In conclusion, our results demonstrate that maternal HIV infection was associated with reduced levels of antibodies against a broad range of pathogens and vaccine antigens in cord blood. Part of this reduction in antibody levels was due to altered antibody levels in the mother, which are the main determinants of cord blood levels, but HIV-infection also diminished transplacental transfer of antibodies. Importantly, IgG1 was the most affected by maternal HIV infection but, depending on the pathogen, other subclasses were also affected. *P. falciparum* exposure also reduced the levels and transfer of some antibodies, although overall the effect was lower than HIV infection. In future studies, the clinical impact of the reduced placental transfer of antibodies caused by HIV infection and *P. falciparum* exposure will be assessed. Our findings are important for effective maternal immunization strategies and for newborn and infant's health.

## Data Availability Statement

The raw data supporting the conclusions of this article will be made available by the authors upon request.

## Ethics Statement

The studies involving human participants were reviewed and approved by Comitè Ètic d'Investigació Clínica (CEIC, Hospital Clínic, UB), Spain, Comité Nacional de Bioética (CNBS), Mozambique. Written informed consent to participate in this study was provided by the participants' legal guardian/next of kin.

## Author Contributions

SA, CD, and GM wrote the first draft of the manuscript, conceived the immunological study, the experimental design, and interpreted the data. CD, GM, RA, and SA designed the analysis and selection of the antigens. SA and MV performed the antibody Luminex assay. SA and MC produced antigens. EA provided the MSP1_42_ protein. GR-O and MV-S performed the statistical analysis. AM, CD, and GM designed the immunology study ancillary to the clinical trials. MM, RB, and CJ processed the samples. PC and LF-S performed the PCR. RG, MR, JA, EM, AV, ES, and CM designed and enrolled participants in the clinical trials. AN was the clinical trial data manager. JA was the clinical trial statistician. RA, MC, RG, CM, GR-O, and MV-S contributed to the write up of the manuscript. All authors reviewed and approved the manuscript.

## Conflict of Interest

The authors declare that the research was conducted in the absence of any commercial or financial relationships that could be construed as a potential conflict of interest.

## Supplementary Material

Supplementary Figure 1 IPTp trial profile.

Supplementary Figure 2 Cord blood antibody levels in HIV-positive women taking ART. Radar charts representing the medians of each analyte antibody cord levels in HIV-positive women who started ART before pregnancy, at recruitment or were not taking ART for IgG **(A)**, IgG1 subclass **(B)**, IgG2 **(C)**, IgG3 **(D)**, and IgG4 **(E)**. Cord blood levels were compared by Kruskal-Wallis test and p-values were adjusted for multiple testing by the Benjamini-Hochberg approach (False Discovery Rate 5%). Statistically significant differences between ART before pregnancy, ART at recruitment and no ART are highlighted with an asterisk. ^**^*p* < 0.01, ^*^*p* < 0.05. ART at recruitment is represented in green, ART before pregnancy is represented in blue and no ART is represented in red.

Supplementary Figure 3 Difference of IgG and IgG subclass levels in cord blood levels of HIV-infected women. Forest plots show the effect (in percentage) of placental malaria on cord blood levels of IgG and IgG subclasses, for all the antigens tested, when placental malaria was included in multivariable models with maternal antibody levels. The differences in percentage correspond to beta-transformed values (%) that were calculated from the beta values obtained in the multivariable models. Beta transformed values (%) are displayed when raw *p*-values are significant. Asterisks are shown when adjusted *p*-values by Benjamini-Hochberg are significant (False Discovery Rate 5%). ^**^*p* < 0.01, ^*^*p* < 0.05.

Supplementary Figure 4 Antibody placental transfer in HIV-positive women taking ART. Radar charts representing the medians of each analyte antibody cord/mother ratio in HIV-positive who started ART before pregnancy, at recruitment or were not taking ART for IgG **(A)**, IgG1 subclass **(B)**, IgG2 **(C)**, IgG3 **(D)**, and IgG4 **(E)**. Ratios were compared by Kruskal-Wallis test and *p*-values were adjusted for multiple testing by the Benjamini-Hochberg approach (False Discovery Rate 5%). Statistically significant differences between ART before pregnancy, ART at recruitment and no ART are highlighted with an asterisk. ^*^*p* < 0.05. ART at recruitment is represented in green, ART before pregnancy is represented in blue and no ART is represented in red.

Supplementary Figure 5 Difference of IgG and IgG subclass placental transfer in HIV-infected women. Forest plots show the effect (in percentage) of placental malaria on transplacental transfer of IgG and IgG subclasses, for all the antigens tested, when placental malaria was included in multivariable models. The differences in percentage correspond to beta-transformed values (%) that were calculated from the beta values obtained in the multivariable models. Beta transformed values (%) are displayed when raw *p*-values are significant. Asterisks are shown when adjusted *p*-values by Benjamini-Hochberg are significant (False Discovery Rate 5%). ^*^*p* < 0.05.

Supplementary Material 1 Cord blood levels and placental transfer of antibodies univariable models.
