# Supplementary figures and images for: Reduced Placental Transfer of Antibodies Against a Wide Range of Microbial and Vaccine Antigens in HIV-Infected Women in Mozambique
Source: Front Immunol. 2021 Mar 3;12:614246. doi: 10.3389/fimmu.2021.614246 (PMC7965965; doi:10.3389/fimmu.2021.614246)

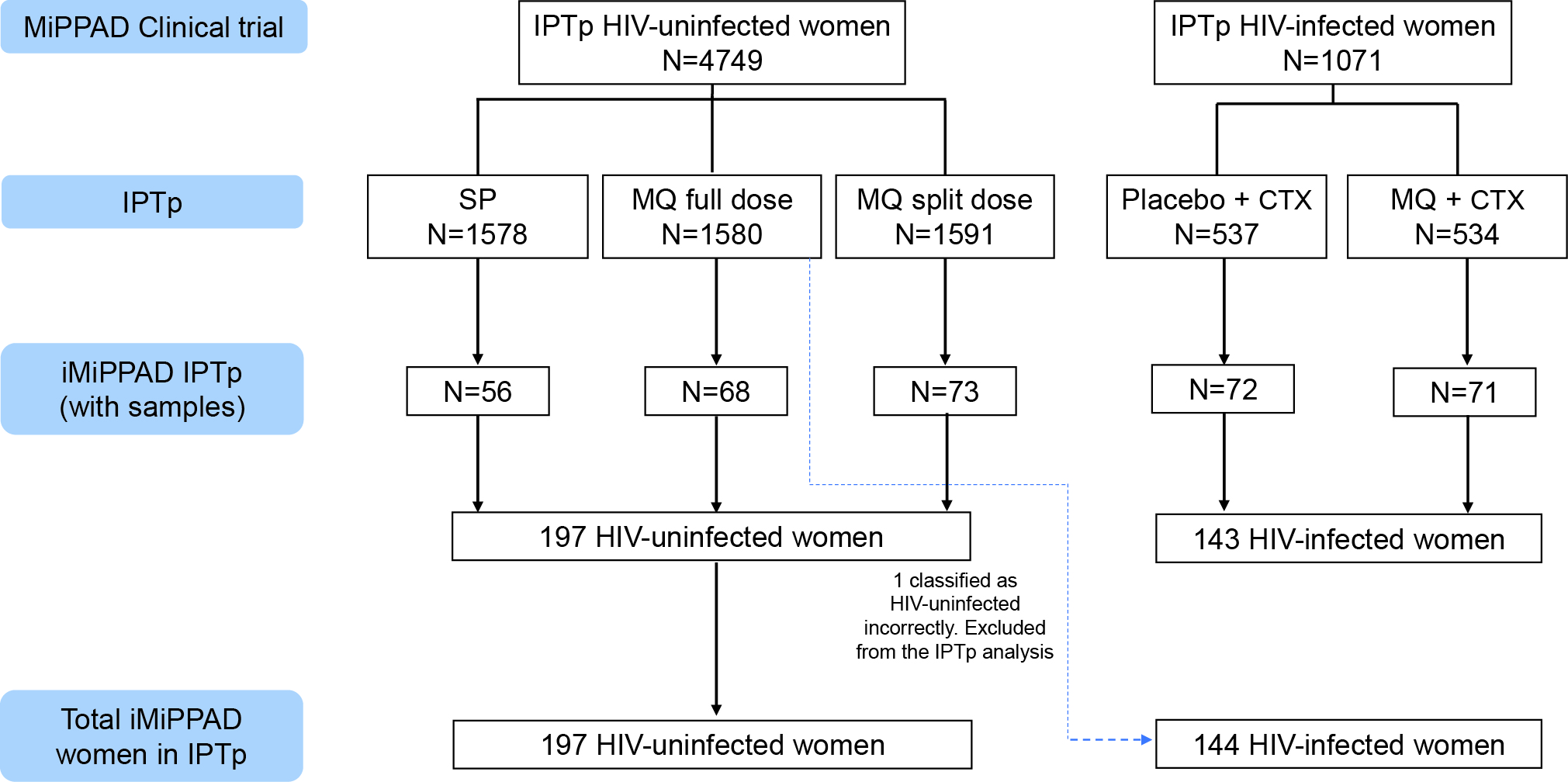

Supplement: Supplementary Figure 1 — IPTp trial profile. [file Image_1.JPEG]

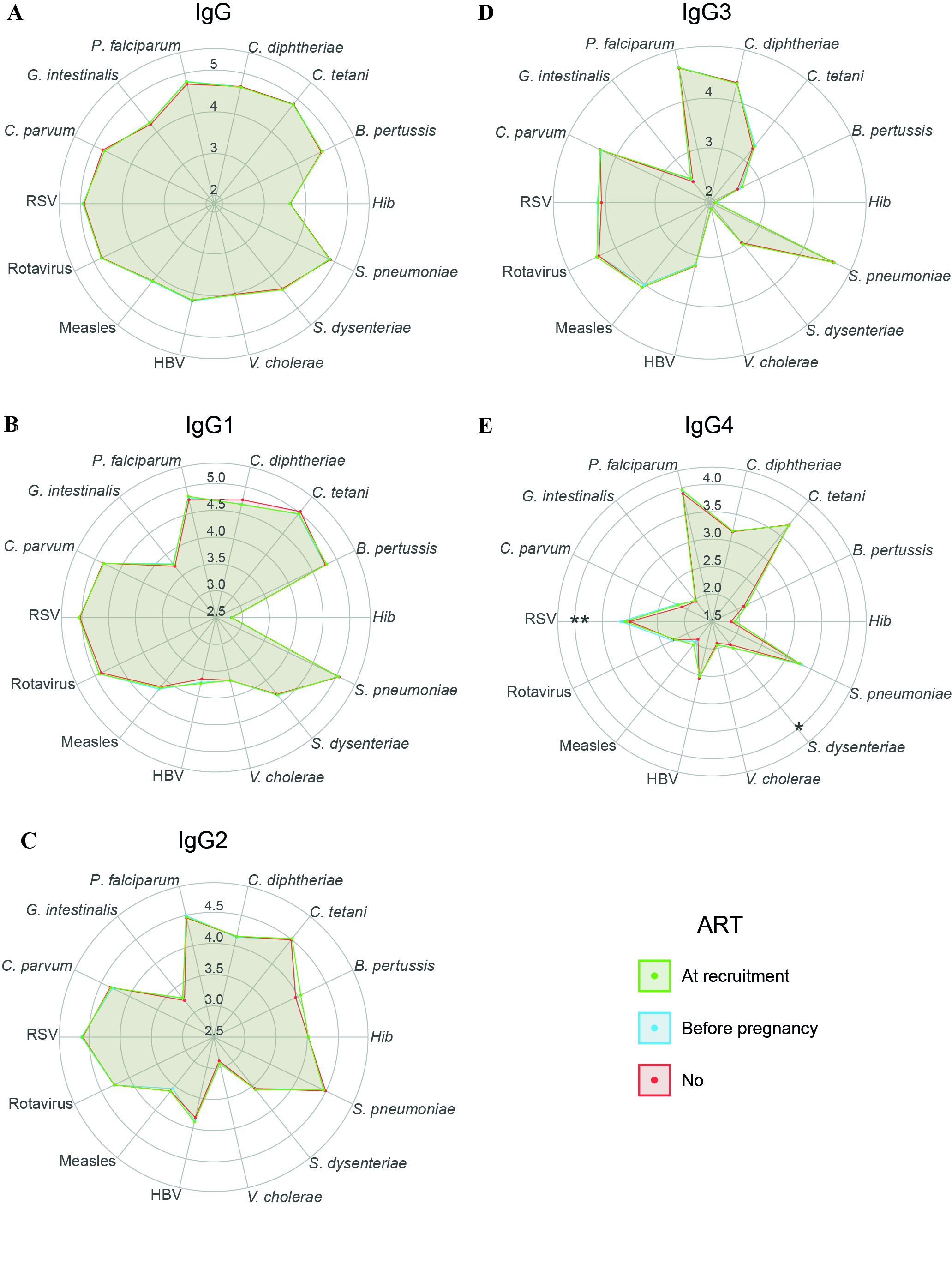

Supplement: Supplementary Figure 2 — Cord blood antibody levels in HIV-positive women taking ART. Radar charts representing the medians of each analyte antibody cord levels in HIV-positive women who started ART before pregnancy, at recruitment or were not taking ART for IgG (A), IgG1 subclass (B), IgG2 (C), IgG3 (D), and IgG4 (E). Cord blood levels were compared by Kruskal-Wallis test and p-values were adjusted for multiple testing by the Benjamini-Hochberg approach (False Discovery Rate 5%). Statistically significant differences between ART before pregnancy, ART at recruitment and no ART are highlighted with an asterisk. **p < 0.01, *p < 0.05. ART at recruitment is represented in green, ART before pregnancy is represented in blue and no ART is represented in red. [file Image_2.JPEG]

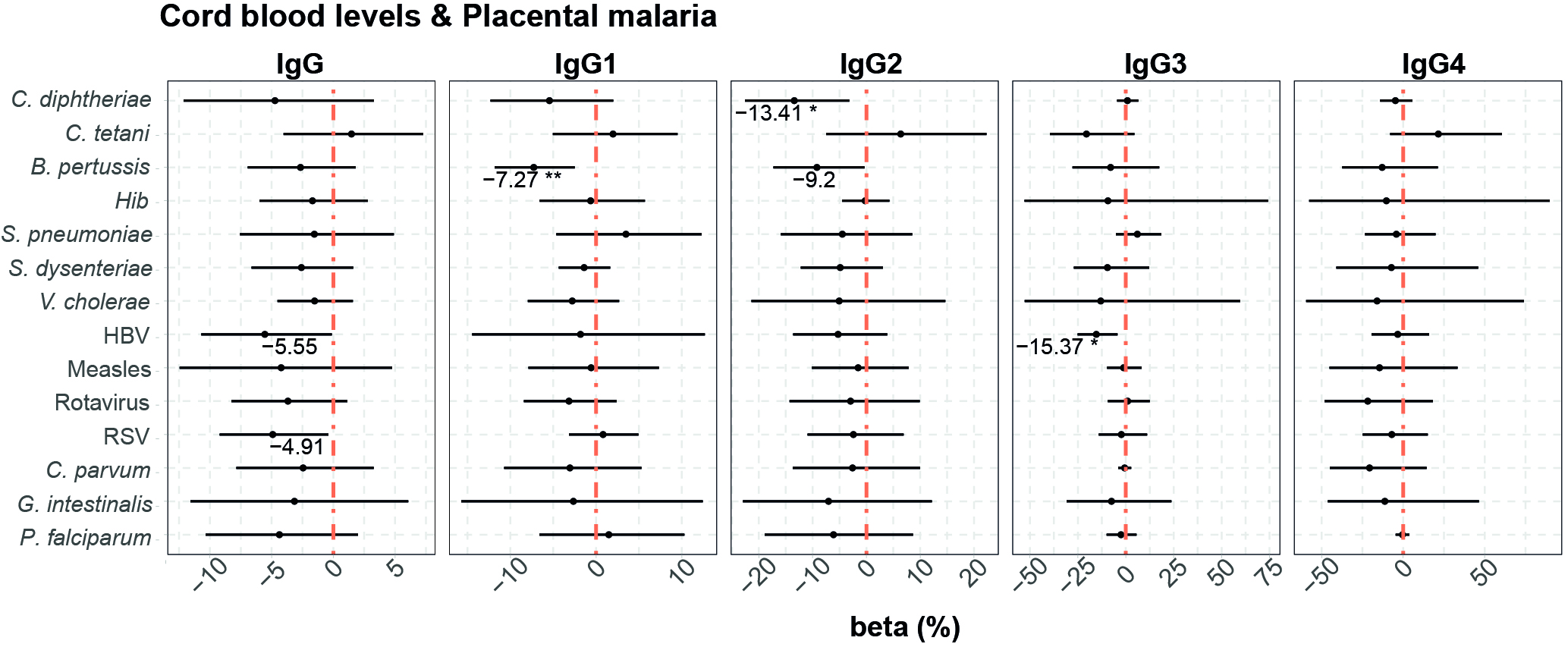

Supplement: Supplementary Figure 3 — Difference of IgG and IgG subclass levels in cord blood levels of HIV-infected women. Forest plots show the effect (in percentage) of placental malaria on cord blood levels of IgG and IgG subclasses, for all the antigens tested, when placental malaria was included in multivariable models with maternal antibody levels. The differences in percentage correspond to beta-transformed values (%) that were calculated from the beta values obtained in the multivariable models. Beta transformed values (%) are displayed when raw p-values are significant. Asterisks are shown when adjusted p-values by Benjamini-Hochberg are significant (False Discovery Rate 5%). **p < 0.01, *p < 0.05. [file Image_3.JPEG]

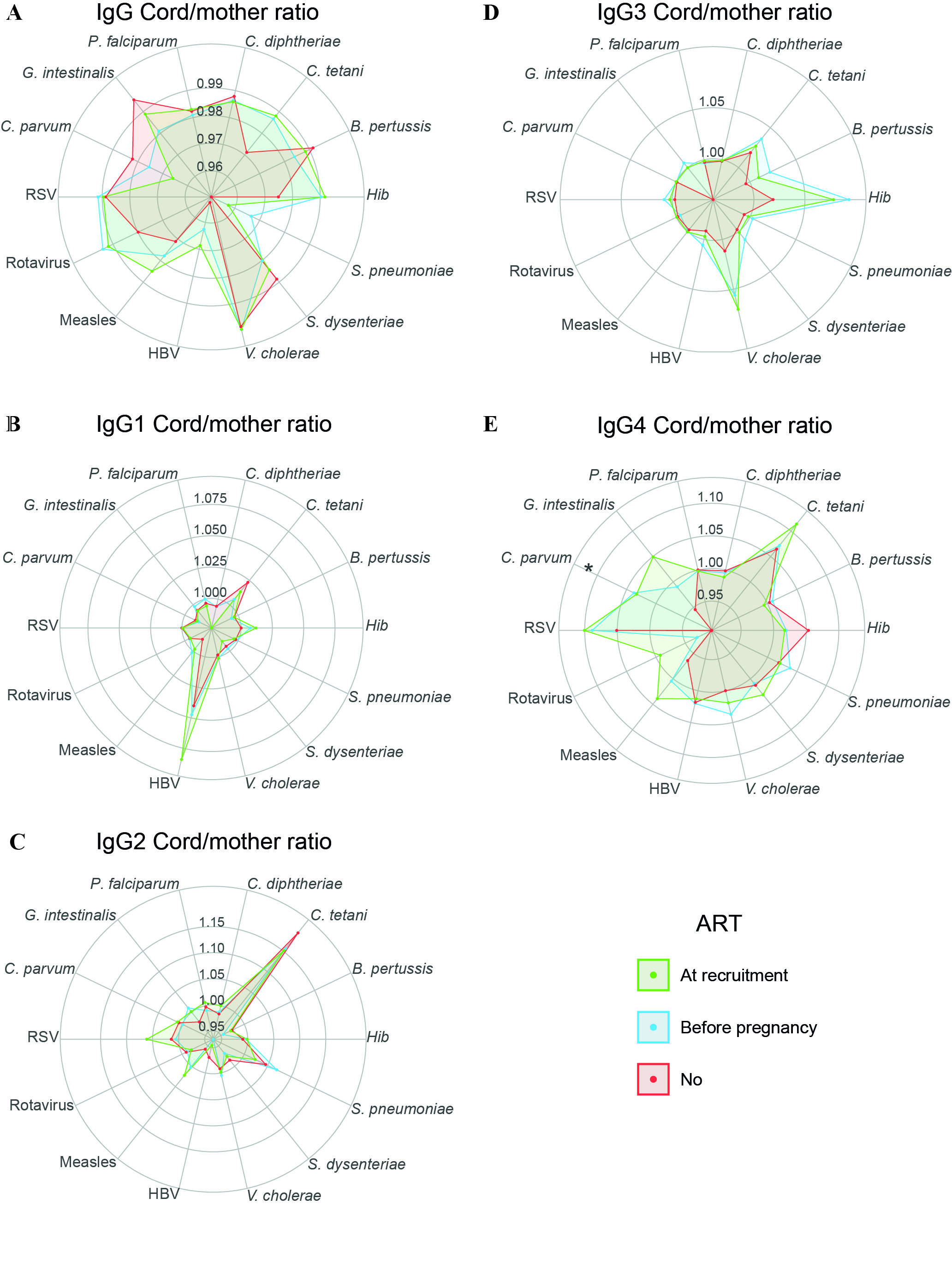

Supplement: Supplementary Figure 4 — Antibody placental transfer in HIV-positive women taking ART. Radar charts representing the medians of each analyte antibody cord/mother ratio in HIV-positive who started ART before pregnancy, at recruitment or were not taking ART for IgG (A), IgG1 subclass (B), IgG2 (C), IgG3 (D), and IgG4 (E). Ratios were compared by Kruskal-Wallis test and p-values were adjusted for multiple testing by the Benjamini-Hochberg approach (False Discovery Rate 5%). Statistically significant differences between ART before pregnancy, ART at recruitment and no ART are highlighted with an asterisk. *p < 0.05. ART at recruitment is represented in green, ART before pregnancy is represented in blue and no ART is represented in red. [file Image_4.JPEG]

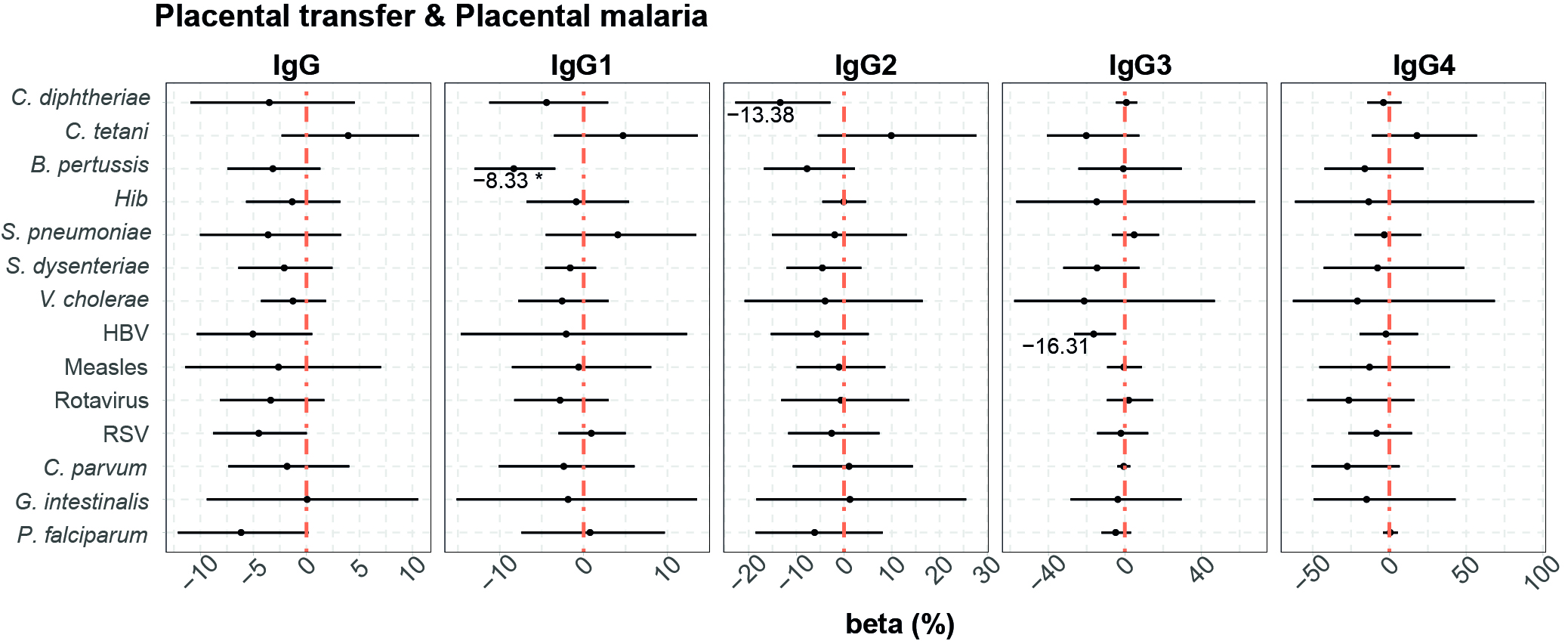

Supplement: Supplementary Figure 5 — Difference of IgG and IgG subclass placental transfer in HIV-infected women. Forest plots show the effect (in percentage) of placental malaria on transplacental transfer of IgG and IgG subclasses, for all the antigens tested, when placental malaria was included in multivariable models. The differences in percentage correspond to beta-transformed values (%) that were calculated from the beta values obtained in the multivariable models. Beta transformed values (%) are displayed when raw p-values are significant. Asterisks are shown when adjusted p-values by Benjamini-Hochberg are significant (False Discovery Rate 5%). *p < 0.05. [file Image_5.JPEG]
